# Going Beyond the Millennium Ecosystem Assessment: An Index System of Human Well-Being

**DOI:** 10.1371/journal.pone.0064582

**Published:** 2013-05-22

**Authors:** Wu Yang, Thomas Dietz, Daniel Boyd Kramer, Xiaodong Chen, Jianguo Liu

**Affiliations:** 1 Center for Systems Integration and Sustainability, Department of Fisheries and Wildlife, Michigan State University, East Lansing, Michigan, United States of America; 2 Environmental Science and Policy Program, Department of Sociology and Animal Studies Program, Michigan State University, East Lansing, Michigan, United States of America; 3 James Madison College and Department of Fisheries and Wildlife, Michigan State University, East Lansing, Michigan, United States of America; 4 Department of Geography, University of North Carolina, Chapel Hill, North Carolina, United States of America; MESC, University of South Alabama, United States of America

## Abstract

Understanding the linkages between ecosystem services (ES) and human well-being (HWB) is crucial to sustain the flow of ES for HWB. The Millennium Ecosystem Assessment (MA) provided a state-of-the-art synthesis of such knowledge. However, due to the complexity of the linkages between ES and HWB, there are still many knowledge gaps, and in particular a lack of quantitative indicators and integrated models based on the MA framework. To fill some of these research needs, we developed a quantitative index system to measure HWB, and assessed the impacts of an external driver – the 2008 Wenchuan Earthquake – on HWB. Our results suggest that our proposed index system of HWB is well-designed, valid and could be useful for better understanding the linkages between ES and HWB. The earthquake significantly affected households' well-being in our demonstration sites. Such impacts differed across space and across the five dimensions of the sub-index (i.e., the basic material for good life, security, health, good social relations, and freedom of choice and action). Since the conceptual framework is based on the generalizable MA framework, our methods should also be applicable to other study areas.

## Introduction

Understanding the linkages between ecosystem services (ES) and human well-being (HWB) is crucial to sustain the flow of ES for HWB [1]. The Millennium Ecosystem Assessment (MA) was intended to provide a state-of-the-art synthesis of such knowledge. ES are defined as the benefits human directly and indirectly obtained from ecosystems [1], [2]. The MA suggested that ecosystems provide services that are of importance for improvements of HWB at multiple scales. These services range from provisioning services such as clean water, food, and forest products, through regulating services such as flood control, soil retention, and air purification, to cultural services such as ecotourism, aesthetic appreciation, and a sense of place [1]. However, during the past five decades, such improvements of HWB were achieved at escalating costs due to the decline or degradation of more than 60% of ES across the globe. This decline or degradation in ES may increase the risks of nonlinear or abrupt changes, and may lead to further marginalization of some groups of people [1].

Although the MA is a monumental work, the linkages between ES and HWB are complex and remain poorly understood [3], [4], [5]. There are four major challenges in developing better understanding of such linkages. First, HWB itself is an evolving and complex concept [3]. It is difficult to provide a universally acceptable definition of HWB. In the MA, HWB has five constituents: the basic material for a good life, security, health, good social relations, and freedom of choice and action [1]. Second, ES substantially, but not exclusively, contribute to HWB. We interpret MA's definition of HWB as the satisfaction of human needs [6] to achieve a state of being well (i.e., healthy, happy, and prosperous), both physically and mentally. While ES substantially satisfy many human needs [1], there are many other influences on well-being, such as personal factors (e.g., personality, self-expectations), demographic factors (e.g., age, and gender), institutional factors (e.g., legal frameworks in which one lives), life experience (e.g., traumatic or disruptive events), and other contextual factors that may affect the subjective feelings of humans [7], [8]. These factors may be affected by ES indirectly rather than directly. For example, threat of violent conflicts may lead to a lack of a sense of security and armed conflicts may be the result of degradation of food supply or other natural resources. Third, the concept of ES is also an evolving concept that changes as we develop new understandings of nature. For example, the human society began to appreciate the carbon sequestration capacity of ecosystems only after recognizing that the increasing carbon emission since the Industrial Revolution is leading to problematic global warming [9]. Meanwhile, it has been widely recognized that the “win-win” solutions are rare and often there are trade-offs among different ES that each contributes to HWB [10], [11]. Finally, the linkages between ES and HWB are bidirectional and dynamic across space and time [5]. Even the most simplified version of MA conceptual framework has demonstrated feedback loops among the four components (i.e., indirect drivers, direct drivers, ES, and HWB) [1].

So far there have been relatively few studies quantitatively integrating the four components of the MA conceptual framework to study the linkages between ES and HWB [1], [12], [13]. Existing quantitative indicators and models of ES were designed under other conceptual frameworks for particular sectors (e.g., land use and land cover change, water supply) or to address the intersections between sectors (e.g., biodiversity and land use and land cover change) [5]. Before the MA, measures of HWB mostly focused on the economic, social-psychological, and health dimensions and did not acknowledge ES as driving forces of HWB [1], [14]. These indices include the World Health Organization's Quality of Life measure (WHOQOL), the Genuine Progress Index (GPI), the Happy Planet Index (HPI), the Human Development Index (HDI), the Life Satisfaction Index, and other different indices of Quality of Life (QoL) [15], [16], [17], [18], [19]. Since the MA, it is becoming widely accepted that HWB cannot be separately considered from ES [14], [20], [21]. Furthermore, many quantitative studies of HWB do not cover all five components of HWB in the MA framework, and thus are inappropriate for the integration of the ES and HWB components. Although qualitative measures of ES and HWB are useful for some studies at the local level [13], they are inadequate to overcome the major challenges discussed above. Rather we require indicators suitable for quantitative analyses (e.g., system modeling and simulation, and detailed statistical analysis of causes and effects). Therefore, developing quantitative indicators and models matching the MA framework is a top priority if we are to understand the linkages between ES and HWB [4], [5].

In recent years, a substantial amount of effort has been made to quantify various ES at multiple scales and assess the trade-offs and synergies that occur in both natural ecosystems and constructed/artificial ecosystems [12], [22], [23], [24], [25], [26], [27], [28]. The Natural Capital Project [12], [27] and the Economics of Ecosystems and Biodiversity (TEEB) project [28] are examples of such efforts that have substantially advanced our understanding of these issues. A few recent studies [14], [20], [21], [29], [30], [31] have discussed in detail how ES contribute to HWB and provided some new insights to improve the understanding of the linkages between ES and HWB. For example, Vemuri and Costanza (2006) and Abdallah et al. (2008) examined how different forms of capital, including natural capital, might explain the life satisfaction at the country level [20], [21]. Jordan et al. (2010) provided a conceptual framework to construct a composite index of HWB, including basic human needs, environmental needs, economic measures and happiness [29]. Dietz et al. (2009, 2012) proposed a model of efficient well-being to assess national efficiency in enhancing HWB through the use of different forms of capital [30], [31]. Summers et al. (2012) comprehensively reviewed the components of HWB with an emphasis on the contribution of ES [14]. However, relatively less attention has been paid to developing quantitative indicators and models of HWB based on the MA framework, nor has there been much work on empirically integrating HWB indicators with indicators and/or models of ES. For instance, quantitatively we know little of how changes in ES, human use of ES, and/or dependence on ES may affect HWB, nor how different population groups have been affected by changes in ES and how have they in turn responded [4], [5], [32]. While theories of how human activities drive environmental changes are progressing steadily, the understanding of how environmental changes may affect humans lag far behind [30].

In response to some of these research needs, in this study, we attempt to (1) develop a new index system to quantify HWB based on the MA framework and (2) empirically demonstrate the application of the index through assessing the impacts of the 2008 Wenchuan Earthquake on HWB.

## Methods

### Ethics statement

We obtained the permission from the Wolong Administration Bureau of Wolong Nature Reserve for conducting household surveys inside the reserve and outside the reserve at Sanjiang Township through the Sanjiang Conservation Station. Since many of our interviewees are not literate or have very low level of education, a verbal consent process was used. We first read the verbal consent script to the selected interviewees. Once they agreed, we then continued to interview them. If consent was not obtained, we did not collect any further information from that interviewee and switched to the next selected interviewee. The survey instruments, verbal consent process, and script were approved by the Institutional Review Board of Michigan State University (http://www.humanresearch.msu.edu/).

### Development of the human well-being index (HWBI) system

We developed a new instrument to measure HWB based on the MA conceptual framework (see the full instrument in the Supporting Information: Data and Instrument File S1). To do this, we first reviewed previous literature and selected a list of indicators for each of the five dimensions. Second, we refined the measures from the literature to situate them in the MA framework, in some cases adding new measures. Third, we pre-tested the indicators with respondents from outside of our research samples and revised them. Finally, we examined the internal validity of the items using item-total correlations to check if any item is inconsistent with the average response across all items. The final list of indicators, the specific asked questions, and the results of internal validity checks are shown in Table S1. Throughout the instrument development process, we followed standard guidelines for using multiple indicators to develop measures of composite variables [33], [34]. Specifically, we pretested the wording to ensure each indicator measured a single, observable outcome. We used positive nomenclature for all the wording of indicators because the technical literature has shown that ratings on negatively worded items or indicators are significantly less reliable than those positively worded [34], [35], [36]. The response to each item was measured with a five category Likert-style scale.

After preliminary reliability analysis, we used Confirmatory Factor Analysis (CFA) to construct the overall index and sub-indices. CFA addresses several problems in this type of data analysis. First, it allows us to avoid the pitfall of assuming individual items are identical with the underlying theoretical variables [37]. CFA allows the boundaries distinguishing the five MA dimensions to be fluid and is open to the possibility that some items may tap multiple underlying variables. For example, a higher satisfaction with housing condition may not only reflect a higher satisfaction with the adequacy of material goods but also a stronger feeling of safety. Second, CFA is a special form of structural equation modeling that handles both the measurement model, that is, the relationship between indicators (or observed measures) and factors (or latent variables), and the casual model linking latent variables to each other and to observed variables [33]. Unlike traditional methods (e.g., principal component analysis), CFA handles easily both the situations of multiple indicators for one factor and one indicator for multiple factors. Third, results of CFA can provide compelling evidence for construct validity [33]. Finally, CFA, unlike traditional methods, allows for hypothesis tests regarding unequal contributions of indicators to the measured factors, minimizes the problem of non-normal and non-continuous distributions of indicators, and adjusts for measurement errors [33], [34].

### Description of the demonstration sites

**Figure 1:**
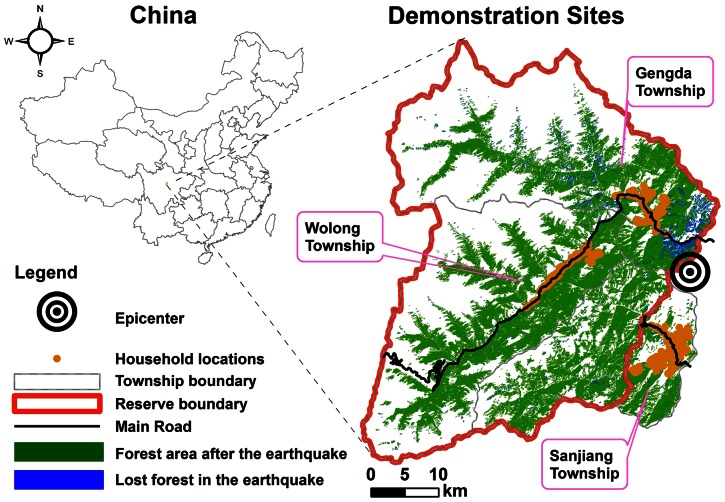
Wolong Nature Reserve and adjacent Sanjiang Township in Wenchuan County, Sichuan Province, southwestern China.

There are four reasons we chose the Wolong Nature Reserve (WNR) and the adjacent Sanjiang Township (SJT) in Wenchuan County of Sichuan Province, southwestern China (Fig. 1) as our sites to demonstrate the utility of our approach. First, they are sites of great ecological importance. Both WNR and SJT belong to the Sichuan Giant Panda Sanctuaries of the United Nations Educational, Scientific and Cultural Organization (UNESCO) World Heritage system. The Sanctuary was established in 2006 to promote the conservation of the giant panda habitat [38]. It also has been classified as one of the world's top 25 Biodiversity Hotspots [39] and one of the Global 200 Eco-regions defined by the World Wildlife Fund [40]. Second, human residents settled the area hundreds of years before the establishment of the sanctuaries and developed ways of life adapted to the local environment. Local residents' well-being substantially depends on many ES. Because human and natural systems are coupled as a result of the current situation as well as a long history [41], [42], successful conservation of the giant panda habitat and associated ecosystems and services will not be achieved if local residents' well-being is ignored. Third, the destructive Wenchuan Earthquake provides a dramatic, if tragic, natural experiment to examine the impacts on HWB. Finally, during the past 18 years, our research team has been working in WNR and has collected extensive data both before and after the earthquake. These formal data are matched with accumulated local knowledge of this area that helped us to design our surveys and interpret our results.

WNR is approximately 2000 km^2^ with approximately 4,900 local rural residents from about 1200 households. SJT is 491 km^2^ of which 344 km^2^ is enclosed in WNR, and all local residents now live in the remaining 147 km^2^ zone outside WNR (Fig. 1). SJT has approximately 4000 local rural residents distributed across 1100 households. The majority of households at WNR and SJT earn their livelihood mainly through agricultural activities (e.g., growing maize and vegetables, raising livestock, and collecting materials for traditional Chinese Medicine), and partly through temporary local jobs (e.g., road construction), small tourism businesses (e.g., selling souvenirs), and migrant work in the cities outside the local area [43].

The epicenter of the devastating 2008 Wenchuan Earthquake (M_s_ 8.0 or M_w_ 7.9) was close to our demonstration sites (Fig. 1). The earthquake caused tremendous socioeconomic and ecological impacts. By September 25, 2008 it was reported that 69,227 people died, 374,643 were injured, 17,923 were missing, and a total of 1,486,407 victims were evacuated and temporally resettled [44]. Rough estimates of the direct and indirect economic losses (e.g., damages to infrastructure, croplands, and tourism) were over one trillion yuan [45]. The earthquake also has caused huge impacts to ecosystems and wildlife habitat. Approximately 122,136 ha (3.40% of the total area of natural ecosystems) were affected including 97,748 ha of forest, 18,021 ha of shrub, 4919 ha of meadow, 1157 ha of barren land, 242 ha of water bodies, and 50 ha of snow-covered land [46]. Approximately 65,584 ha of panda habitat (5.92% of the total panda habitat) were damaged with 34,737 ha and 30,847 ha distributed inside and outside nature reserves respectively [46]. Although both WNR and SJT were affected by the earthquake, the impacts at SJT were less severe than those at WNR. Forty-eight local residents of WNR and seven of SJT died in the earthquake. Several additional hundreds of workers and passengers died along the road within the reserve. Infrastructure such as the roads, residential houses, schools, hospitals, and tourism facilities were destroyed at WNR but were less damaged at SJT. In fact, after the earthquake, since the main road of WNR was blocked while the road of SJT was accessible, many people inside WNR fled using trails to SJT. The variation in earthquake impacts between WNR and SJT allows us to examine the differential effects of the 2008 Wenchuan Earthquake on HWB.

### Household surveys

During the summer of 2010 we randomly sampled approximately 15% of local households both inside and outside the reserve for a total of 169 households at WNR and 157 households at SJT. Because our past experience in this area suggests that household heads or their spouses are usually the decision makers about household affairs and thus most familiar with the questions we were asking [47], we chose them as interviewees. For the collection of retrospective data, we followed standard practices of life history calendars to enhance respondents' recall accuracy [48], [49]. Before conducting the formal household interviews, we first explained the meaning of each indicator to our local field assistants, and pretested and revised the survey instrument with households outside of our sample area. Because most of the interviewees were farmers with low literacy, we implemented the interviews face-to-face using local languages. The dataset and instrument used for this study are provided in the Supporting Information (Data and Instrument File S1).

## Results

### Internal consistency of the HWBI system

The combination of indicators we used appears to be an appropriate measure of HWB. The item-total correlations for each indicator are reasonably strong, ranging from 0.30 to 0.74 before and 0.33 to 0.75 after the earthquake (Table S1). The Cronbach's alpha values are high – 0.92 and 0.91 for before and after the earthquake, respectively. Moreover, the deletion of any of the indicators reduces the value of alpha for both before and after the earthquake. Thus these items appear to have reasonable internal validity.

### CFA results of the HWBI system

Model fit statistics show that the goodness-of-fit of our CFA is high regardless of the criterion used (Table 1). The ratio of Chi-Square to the degrees of freedom (df) is 1.6, which is lower than the commonly used maximum of 3 as a criterion for adequate fit [50]. The Comparative Fit Index (CFI) and Tucker-Lewis Index (TLI) are 0.976 and 0.971 respectively, again indicating good fit. Both the Root Mean Square Error of Approximation (RMSEA) and Standardized Root Mean Square Residual (SRMR) are lower than 0.05. No modification indices are above the default threshold of 10 suggested by Muthen and Muthen [51]. In addition to the overall fit statistics, the significance tests of coefficients for each path and the test of significance for each path's contribution to model fit also show high goodness-of-fit and construct validity for each indicator (p<0.05, Table S2).

**Table 1:** Summary of model fit information for the confirmatory factor analysis.

Fit statistics	Value
Ratio of Chi-Square to df (χ^2^/df)	1.6
CFI (Comparative Fit Index)	0.976
TLI (Tucker-Lewis Index)	0.971
RMSEA (Root Mean Square Error of Approximation)	0.030
SRMR (Standardized Root Mean Square Residual)	0.035

Notes: ^***^ p<0.001. The Chi-square value is for the MLR estimator (maximum likelihood estimation with robust standard errors) in Mplus, which is not used for Chi-square difference testing in the regular way. No modification indices are above the default threshold of 10 in Mplus. All observed variables and latent variables are tested to significantly (p<0.05) contribute to model fit.

**Figure 2:**
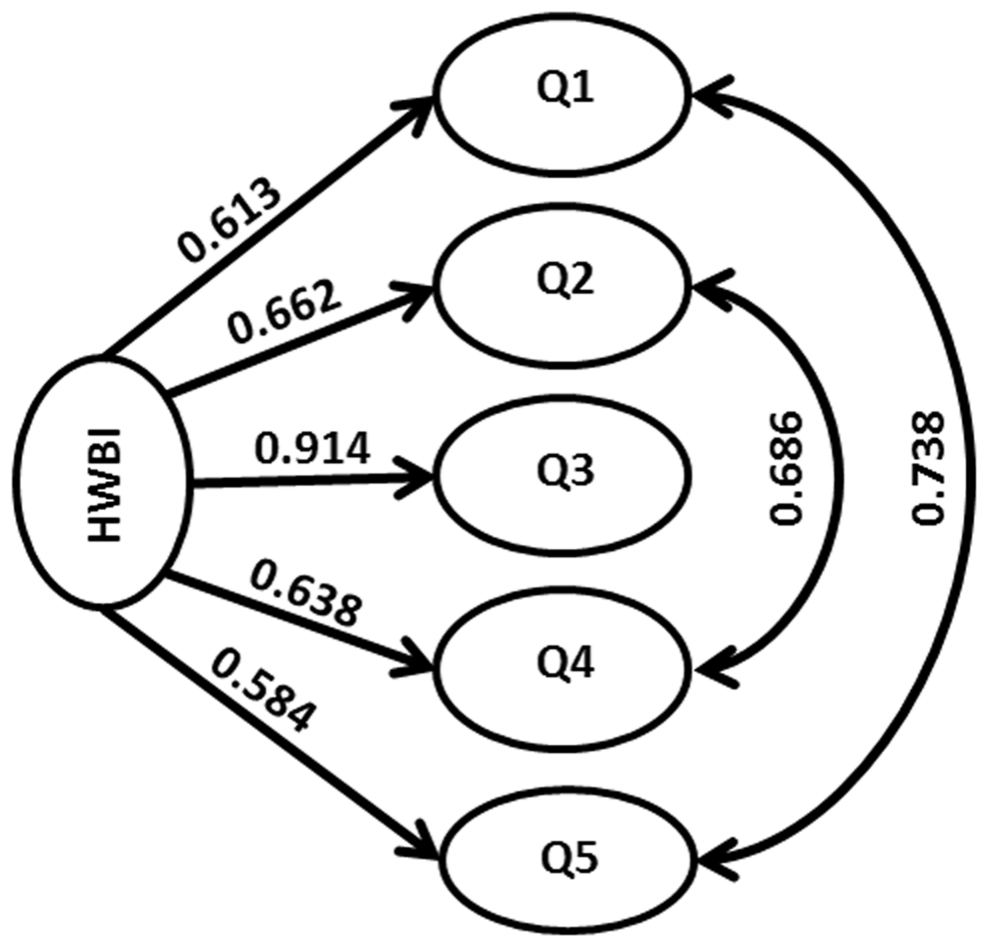
Path diagram of the confirmatory factor analysis model for HWBI. HWBI: Human well-being index; Q1: Basic material for good life; Q2: Security; Q3: Health; Q4: Good social relations; Q5: Freedom of choice and action. Single-headed arrows indicate the direction of causal influence, and double-headed arrows represent covariance between two latent variables. Number on each path represents the standardized coefficient estimated by the confirmatory factor analysis model. Paths of the structural model are not shown here. Detailed description of observed indicators and model results are shown in Table S1 and S2.

Our results also suggest that all five latent variables representing the five dimensions of HWB have significant coefficients and significantly contribute to the model fit (p<0.001, Fig. 2 and Table S2). These results are consistent with the MA structure of five different dimensions of HWB.

However, our results (Fig. 2, Table S2) also suggest that the dimension of basic material for good life is significantly positively associated with the dimension of freedom of choice and action (p<0.001), and the dimension of security is significantly positively associated with the dimension of good social relations (p<0.001). This evidence suggests that the five dimensions are not fully independent (as we suspected would be the case while conceptualizing the five dimensions), and thus it is appropriate to use CFA instead of the principal component analysis that usually assumes orthogonality of latent variables.

### Impacts of the earthquake on HWBI

Table 2 provides descriptive statistics of sub-indices and overall HWBI both inside and outside the reserve before and after the earthquake. Our results show that overall HWBI and sub-indices inside the reserve were all significantly higher than those outside the reserve both before and after the earthquake (Table 2). However, the impacts of the earthquake on overall HWBI and sub-indices differed from inside to outside the reserve, as indicated by the interaction term in the regressions estimating the effects of the earthquake (Table 3). All the coefficients of interaction terms between the pre-earthquake value and the research site were negative, and all but the term for good social relations were statistically significant (Table 3). It appears that the decreases in the sub-indices and overall HWBI inside the reserve were larger than those outside the reserve. Of the coefficients of the pre-earthquake index values, only the sub-index of freedom of choice and action and overall HWBI were significant (p<0.05) and both are positive. This suggests that households with higher freedom of choice and action or overall HWBI pre-earthquake decreased less in freedom of choice and action and in overall HWBI – high values seemed to buffer against the adverse impacts of the earthquake.

**Table 2:** Descriptive statistics of sub-indices and overall HWBI both inside and outside the reserve before and after the earthquake.

Human well-being	Before earthquake			After earthquake		
	Inside	Outside	t value	Inside	Outside	t value
Basic material for good life (Q1)	0.461 (0.018)	0.239 (0.014)	9.870^***^	0.439 (0.018)	0.267 (0.017)	7.039^***^
Security (Q2)	0.692 (0.010)	0.463 (0.015)	12.459^***^	0.603 (0.013)	0.445 (0.018)	7.002^***^
Health (Q3)	0.668 (0.010)	0.467 (0.013)	12.234^***^	0.589 (0.012)	0.403 (0.016)	9.094^***^
Good social relations (Q4)	0.685 (0.010)	0.465 (0.014)	12.623^***^	0.642 (0.012)	0.474 (0.017)	8.111^**^
Freedom of choice and action (Q5)	0.387 (0.018)	0.140 (0.013)	11.006^***^	0.364 (0.018)	0.151 (0.016)	8.946^***^
Overall HWBI	0.640 (0.009)	0.422 (0.013)	13.746^***^	0.566 (0.012)	0.375 (0.016)	9.646^***^

Notes: Numbers outside and inside parentheses are means and standard errors for changes of overall indices and sub-indices, respectively. The numbers of observations are 169 and 157 inside and outside the reserve both before and after the earthquake, respectively. The overall index and sub-indices are respectively normalized into the range from 0 to 1 using the maximum-minimum normalization method.

**Table 3:** Impacts of the earthquake on sub-indices and overall HWBI inside and outside the reserve.

Independent variables	Q1	Q2	Q3	Q4	Q5	Overall HWBI
Pre-earthquake index	1.082 (0.055)	1.029 (0.043)	1.031 (0.048)	1.048 (0.043)	1.111^*^ (0.049)	1.104^*^ (0.067)
Site (0: outside; 1: inside reserve)	−0.002 (0.014)	0.032 (0.044)	0.080^†^ (0.044)	−0.012 (0.036)	0.019 (0.012)	0.067^†^ (0.036)
Pre-earthquake index × Site	−0.145^*^ (0.060)	−0.159^*^ (0.071)	−0.151^*^ (0.075)	−0.074 (0.060)	−0.207^**^ (0.060)	−0.182^**^ (0.064)
Constant	0.009	−0.031	−0.079^**^	−0.013	−0.004	−0.091^***^

Notes: Dependent variables are corresponding sub-indices or overall HWBI post-earthquake respectively. Numbers outside and inside parentheses are coefficients and standard errors, respectively. The number of total observation is 326, including 169 and 157 observations inside and outside the reserve, respectively. ^†^p<0.1; ^*^p<0.05; ^**^p<0.01; ^***^p<0.001.

## Discussion

We proposed a HWBI system based on the MA framework and empirically demonstrated its construct validity. Further, the difference in the effects of the earthquake on HWB indices between households outside and inside the reserve is evidence that the observed impacts on HWB are consistent with what we would expect as a result of the earthquake and short-term post-earthquake situation. So we believe we have a strong case for both the internal and external validity of our proposed measure.

Compared to outside the reserve, the larger decreases in the overall index and sub-indices of HWB inside the reserve are probably because pre-earthquake the overall index and sub-indices were higher inside the reserve and because there were more severe damages, especially destruction to the main road connecting the reserve with the outside world. The significant decreases in sub-indices for security and health indicate that the earthquake caused not only physical damages affecting local households' livelihoods but also had negative impacts on their mental health. Nevertheless, the short-term post-earthquake reconstruction efforts seemed to turn the disaster into opportunities to improve local households' welfare. Outside the reserve, the sub-indices for basic material for good life and freedom of choice and action actually increased significantly (p<0.001 and p<0.05, respectively) after the earthquake. One major reason is undoubtedly post-earthquake road construction. Due to the implementation of the Wenchuan Earthquake Reconstruction Plan [52], road conditions outside the reserve have dramatically improved and all nine villages at SJT now have cement pavement roads connecting to the main road. In contrast to the construction efforts in the STJ, those inside the reserve suffer from frequent post-earthquake natural disasters (e.g., mud-rock flows and landslides) that are less problematic for those outside.

Our results also suggest that households with higher overall HWBI or less freedom of choice and action pre-earthquake were less affected than those with lower indices. These results are consistent with findings from other studies such as the Hurricane Katrina [53]. It is probably because those households with lower overall HWBI or less freedom of choice and action lack adaptive capacity and thus were more vulnerable to the disaster [54]. This pattern holds both inside and outside the reserve. It indicates that it is not caused by different socioeconomic contexts inside and outside the reserve. Unfortunately the post-earthquake reconstruction policy had not addressed this problem by the time of our investigation in the summer of 2010. This suggests that post-earthquake policies should give priority to those households with lower overall HWBI or less freedom of choice and action.

The post-earthquake reconstruction outside the reserve was almost completed by May of 2010 but is still ongoing inside the reserve with completion planned for 2015. At this time it is difficult to predict how the earthquake-induced changes in HWB may in turn affect households' socioeconomic activities and use of ES in the long run. But according to information gained from regular monitoring by the local government and our own field investigation, starting shortly after the earthquake there seemed to be dramatic increases of illegal logging and poaching outside the reserve and increases in poaching inside the reserve. This indicates that post-earthquake reconstruction must consider households' use of and dependence on ES and their interactions with HWB. Priorities should be given to helping local households to build capacity and find alternative income sources that do not harm or offset conservation efforts.

We believe the HWB index systems we developed has some major advantages compared to other approaches that have been proposed. First, our index system is based on the general MA framework and explicitly considers the contribution of ES to HWB. Second, its construct validity has been confirmed empirically in our demonstration sites. Therefore, it could easily be applied to other study areas and across different scales with some modifications if necessary. Third, our index system is developed using CFA techniques that examine the relationships of multiple indicators to multiple factors (i.e., dimensions) and the correlations among different dimensions. This allows a nuanced assessment of the measurement properties of the index and its components. Finally, our index system provides both a composite index and sub-indices. This allows the quantitative examination of the interwoven linkages between different types of ES and different components of HWB. The value of having both aggregate and disaggregate measures was evident when we considered the effects of the earthquake, which differed across sub-indices. However, similar to other composite indices but unlike single question indices, we acknowledge that this advantage is achieved at the costs of more data being needed and a more effort in constructing the measure.

## Conclusions and Implications

We developed an index of HWB based on the MA framework and applied it to a region in which ES are very important for HWB. Our results suggest that our HWBI system has reasonable internal and external validity. Our index was able to detect the Wenchuan earthquake's impact on household well-being, and show that the estimated impacts differed between households inside and outside the reserve as well as across the five dimensions of the sub-index.

Human and natural systems are complex and coupled [41]. Human use of and dependence on ES affects HWB, and changes in HWB may in turn affect human use of and dependence on ES. Our analysis points to some practical implications of this coupling. If post-earthquake reconstruction policies do not adequately address the negative impacts of the earthquake on local households, especially those with less freedom of choice and action and lower overall HWBI, many households may be forced to find alternative income sources including illegal logging and poaching to maintain basic livelihoods.

Our proposed HWBI system seems to be a viable approach and could be useful for further research to better understand the linkages between ES and HWB. We demonstrated the development of the HWBI system and its application in assessing the impacts of 2008 Wenchuan Earthquake on households' well-being. Since the conceptual framework is based on the generalizable MA framework, our methods should also be applicable to other study areas.

## Supporting Information

Data and Instrument File S1 **Dataset and instrument used in this study.**
(ZIP)

Table S1 **The index system for assessing human well-being based on the Millennium Ecosystem Assessment conceptual framework.**
(DOC)

Table S2 **Standardized coefficients of the confirmatory factor analysis for Human Well-Being Index (HWBI).**
(DOC)
