# Supplementary material for: Going Beyond the Millennium Ecosystem Assessment: An Index System of Human Well-Being
Source: PLoS One. 2013 May 22;8(5):e64582. doi: 10.1371/journal.pone.0064582 (PMC3661712; doi:10.1371/journal.pone.0064582)
Supplement: Table S1 — The index system for assessing human well-being based on the Millennium Ecosystem Assessment conceptual framework. (DOC) [file pone.0064582.s002.doc]

Table S1. The index system for assessing human well-being based on the Millennium Ecosystem Assessment conceptual framework.

| Indicator layer | Indicator code and content | *r_i-t_* before earthquake | *r_i-t_* after earthquake |
| --- | --- | --- | --- |
| Affordability to necessary food | Q1.2: Your household can afford enough food with nutrition to keep alive and healthy | 0.64 | 0.66 |
| Affordability to basic facilities and services | Q1.3: Your household can afford to access basic facilities (e.g., television, washer) and services (e.g., transportation) | 0.62 | 0.64 |
| Satisfaction with housing condition | Q1.4: You are satisfied with your housing condition (including size and quality) | 0.50 | 0.40 |
| Overall satisfaction with access to basic goods and services | Q1.5: Overall, you are satisfied with your household’s basic goods and services (e.g., food, clothe, living conditions, transportation) for life | 0.68 | 0.64 |
| Life safety | Q2.1: Your household’s life safety in daily life is secure | 0.59 | 0.46 |
| Property safety | Q2.2: Your household’s property safety in daily life is secure | 0.54 | 0.49 |
| Local crime incidence | Q2.3: The local crime incidence (e.g., theft, robbery, murder, other violent incidents) is low | 0.51 | 0.41 |
| Access to government protection | Q2.4: The police and judicial system is always ready to help | 0.40 | 0.38 |
| Reliability of government protection | Q2.5: The police and judicial system can be trusted | 0.38 | 0.33 |
| Security for resource access | Q2.6: It is safe to access basic goods and services such as food, water, and medicine etc. for life | 0.56 | 0.49 |
| Overall satisfaction with security | Q2.7: Overall, you are satisfied with your household security (e.g., life and property) | 0.66 | 0.59 |
| Physical health | Q3.1: You are satisfied with your household’s physical health (including illness and injury)? | 0.67 | 0.66 |
| Mental health | Q3.2: You are satisfied with your household’s mental health (including stress, depression, and problems with emotions)? | 0.66 | 0.68 |
| Rest | Q3.3: How often your household members do not get enough rest or sleep? (Options: 1. Always; 2. Often; 3. Sometimes; 4. Seldom; 5. Never) | 0.50 | 0.60 |
| Energy for daily life | Q3.4: How often your household members are not healthy or do not have enough energy for everyday life? (Options: 1. Always; 2. Often; 3. Sometimes; 4. Seldom; 5. Never) | 0.59 | 0.63 |
| Emotion | Q3.5: How often do your household members have negative feelings such as blue mood, despair, anxiety, depression? (Options: 1. Always; 2. Often; 3. Sometimes; 4. Seldom; 5. Never) | 0.55 | 0.61 |
| Leisure activities | Q3.6: How often do your household members have the opportunity for leisure activities? (Options: 1. Never; 2. Seldom; 3. Sometimes; 4. Often; 5. Always) | 0.45 | 0.52 |
| Overall satisfaction with health status | Q3.7: Overall, you are satisfied with your household’s health status | 0.66 | 0.70 |
| Close neighborhood | Q4.1: This is a close-knit neighborhood | 0.53 | 0.43 |
| Opportunities of neighborhood interactions | Q4.3: There are many opportunities to meet neighbors and work on solving community problems | 0.31 | 0.37 |
| Cohesion | Q4.6: Suppose someone in your village/neighborhood had something unfortunate happen to them, such as a family member's sudden death, there are always some others would be ready to help | 0.37 | 0.39 |
| Overall satisfaction with social relationship | Q4.7: Overall, you are satisfied with your household’s social relationships with others | 0.38 | 0.36 |
| Affordability to quality and nutritious food | Q5.2: Your household has affordable access to quality and nutritious food for an enjoyable life | 0.73 | 0.74 |
| Affordability to quality healthcare | Q5.3: Your household has affordable access to quality medical care | 0.74 | 0.75 |
| Affordability to quality education | Q5.4: Your household has affordable access to quality education | 0.72 | 0.71 |
| Affordability to quality housing | Q5.5: Your household has affordable access to spacious and quality house | 0.65 | 0.57 |
| Free choice of employment | Q5.6: It is difficult to find a satisfied job | 0.30 | 0.33 |
| Overall satisfaction with freedom of choice and action | Q5.8: Overall, you are satisfied with your freedom of choice and actions | 0.63 | 0.61 |

Notes:

*r_i-t_* : item-test correlation. Cronbach’s α values for standardized items are 0.92 and 0.91 before and after the earthquake, respectively.

Except for response options specified after indicator contents, options for all other indicator contents are designed in the five-category Likert scale (i.e., strongly disagree, mildly disagree, unsure, mildly agree, and strongly agree). All the responses are coded in the order from the lowest score of 1 to the highest score of 5. A higher score represents a higher level of well-being. Actual surveys are conducted through face-to-face interviews using the local language that is easily understandable to interviewees. The full instrument and dataset are attached in the Supporting Information file.
