# Supplementary material for: Going Beyond the Millennium Ecosystem Assessment: An Index System of Human Well-Being
Source: PLoS One. 2013 May 22;8(5):e64582. doi: 10.1371/journal.pone.0064582 (PMC3661712; doi:10.1371/journal.pone.0064582)
Supplement: Table S2 — Standardized coefficients of the confirmatory factor analysis for Human Well-Being Index (HWBI). (DOC) [file pone.0064582.s003.doc]

Table S2. Standardized coefficients of the confirmatory factor analysis for Human Well-Being Index (HWBI).

| Dependent variable | Independent variable | Standardized coefficients | Robust S.E. |
| --- | --- | --- | --- |
| Overall HWBI | Q1 | 0.613^***^ | 0.034 |
|  | Q2 | 0.662^***^ | 0.045 |
|  | Q3 | 0.914^***^ | 0.042 |
|  | Q4 | 0.638^***^ | 0.054 |
|  | Q5 | 0.584^***^ | 0.034 |
| Q1：Basic material for good life | Q1.2 | 0.826^***^ | 0.018 |
|  | Q1.3 | 0.829^***^ | 0.018 |
|  | Q1.4 | 0.235^***^ | 0.043 |
|  | Q1.5 | 0.427^***^ | 0.035 |
|  | Q3.6 | 0.218^***^ | 0.044 |
|  | Q5.2 | 0.657^***^ | 0.080 |
|  | Q5.3 | 0.374^***^ | 0.079 |
| Q2：Security | Q2.1 | 0.617^***^ | 0.035 |
|  | Q2.2 | 0.602^***^ | 0.035 |
|  | Q2.4 | 0.336^***^ | 0.042 |
|  | Q2.5 | 0.314^***^ | 0.042 |
|  | Q2.6 | 0.381^***^ | 0.044 |
|  | Q2.7 | 0.818^***^ | 0.027 |
|  | Q1.4 | 0.262^***^ | 0.044 |
|  | Q1.5 | 0.368^***^ | 0.040 |
|  | Q3.2 | 0.199^***^ | 0.046 |
| Q3：Health | Q3.1 | 0.787^***^ | 0.026 |
|  | Q3.2 | 0.642^***^ | 0.042 |
|  | Q3.3 | 0.618^***^ | 0.033 |
|  | Q3.4 | 0.700^***^ | 0.027 |
|  | Q3.5 | 0.597^***^ | 0.032 |
|  | Q3.7 | 0.826^***^ | 0.021 |
| Q4：Good social relations | Q4.1 | 0.548^***^ | 0.039 |
|  | Q4.3 | 0.300^***^ | 0.041 |
|  | Q4.6 | 0.438^***^ | 0.037 |
|  | Q4.7 | 0.417^***^ | 0.043 |
|  | Q2.3 | 0.533^***^ | 0.035 |
|  | Q3.6 | 0.367^***^ | 0.048 |
| Q5：Freedom of choice and action | Q5.2 | 0.263^***^ | 0.075 |
|  | Q5.3 | 0.553^***^ | 0.080 |
|  | Q5.4 | 0.909^***^ | 0.017 |
|  | Q5.5 | 0.784^***^ | 0.024 |
|  | Q5.6 | 0.274^***^ | 0.041 |
|  | Q5.8 | 0.808^***^ | 0.023 |
|  | Q2.6 | 0.226^***^ | 0.043 |
| Q1 | Q5 | 0.738^***^ | 0.033 |
| Q2 | Q4 | 0.686^***^ | 0.075 |

Notes:

^***^p < 0.001. For description of each code, please refer to Appendix A. Only paths that are theoretically meaningful are added. Only paths with coefficients that are tested to be significant (p < 0.05) are included in the model. Paths between observed indicators are not shown here. The confirmatory factor analysis is constructed using the MLR estimator in Mplus. The number of total observations is 326.
